# Up-regulation of microglial cathepsin C expression and activity in lipopolysaccharide-induced neuroinflammation

**DOI:** 10.1186/1742-2094-9-96

**Published:** 2012-05-20

**Authors:** Kai Fan, Xuefei Wu, Bin Fan, Ning Li, Yongzhong Lin, Yiwen Yao, Jianmei Ma

**Affiliations:** 1 Department of Anatomy, Dalian Medical University, No. 9, West Segment of South Lvshun Road, Dalian, Liaoning, 116044, China; 2 Department of Physiology, Dalian Medical University, No. 9, West Segment of South Lvshun Road, Dalian, Liaoning, 116044, China; 3 Graduate School, Dalian Medical University, No. 9, West Segment of South Lvshun Road, Dalian, Liaoning, 116044, China; 4 General Surgery, Wafangdian Central Hospital, No. 3, Jinluan Road, Wafangdian, Liaoning, 116300, China; 5 Department of Neurology, the First Affiliated Hospital of Dalian Medical University, No. 222, Zhongshan Road, Dalian, Liaoning, 116023, China; 6 Department of Otolaryngology, the First Affiliated Hospital of Dalian Medical University, No. 222, Zhongshan Road, Dalian, Liaoning, 116023, China

**Keywords:** Cathepsin C, Microglia, Lipopolysaccharide, Neuroinflammation

## Abstract

**Background:**

Cathepsin C (Cat C) functions as a central coordinator for activation of many serine proteases in inflammatory cells. It has been recognized that Cat C is responsible for neutrophil recruitment and production of chemokines and cytokines in many inflammatory diseases. However, Cat C expression and its functional role in the brain under normal conditions or in neuroinflammatory processes remain unclear. Our previous study showed that Cat C promoted the progress of brain demyelination in cuprizone-treated mice. The present study further investigated the Cat C expression and activity in lipopolysaccharide (LPS)-induced neuroinflammation *in vivo* and *in vitro.*

**Methods:**

C57BL/6 J mice were intraperitoneally injected with either 0.9% saline or lipopolysaccharide (LPS, 5 mg/kg). Immunohistochemistry (IHC) and *in situ* hybridization (ISH) were used to analyze microglial activation, TNF-α, IL-1β, IL-6, iNOS mRNAs expressions and cellular localization of Cat C in the brain. Nitrite assay was used to examine microglial activation *in vitro*; RT-PCR and ELISA were used to determine the expression and release of Cat C. Cat C activity was analyzed by cellular Cat C assay kit. Data were evaluated for statistical significance with paired *t* test.

**Results:**

Cat C was predominantly expressed in hippocampal CA2 neurons in C57BL/6 J mice under normal conditions. Six hours after LPS injection, Cat C expression was detected in cerebral cortical neurons; whereas, twenty-four hours later, Cat C expression was captured in activated microglial cells throughout the entire brain. The duration of induced Cat C expression in neurons and in microglial cells was ten days and three days, respectively. *In vitro*, LPS, IL-1β and IL-6 treatments increased microglial Cat C expression in a dose-dependent manner and upregulated Cat C secretion and its activity.

**Conclusions:**

Taken together, these data indicate that LPS and proinflammatory cytokines IL-1β, IL-6 induce the expression, release and upregulate enzymatic activity of Cat C in microglial cells. Further investigation is required to determine the functional role of Cat C in the progression of neuroinflammation, which may have implications for therapeutics for the prevention of neuroinflammation-involved neurological disorders in the future.

## Background

Neuroinflammation is associated with many neurodegenerative diseases, including Alzheimer’s disease, Parkinson’s disease, amyotrophic lateral sclerosis, and multiple sclerosis. The ubiquitous pathological changes of neuroinflammation in these diseases include overactivation of glial cells, increased proinflammatory cytokine concentration, increased blood–brain barrier (BBB) permeability, and leukocyte invasion [1-3]. Numerous reports have demonstrated that neuroinflammation contributes to the causation and aggravation of neurodegenerative diseases [4-6]. The inflammatory mediators, such as interleukin-1β (IL-1β), interleukin-6 (IL-6), tumor necrosis factor-α (TNF-α), chemokines and oxygen free radicals are assumed to stimulate signaling pathways in a pathological cascade to result in neurodegenerative diseases [7-14].

Cathepsins, a large group of lysosomal proteases, have been found to participate in the neuroinflammatory processes [15-21]. According to their catalytic mechanism, they are further defined as cysteine (cathepsin B, C, H, L, S, Z, and so on), aspartic (cathepsin D, and so on) and serine proteases (cathepsin G, and so on) [22]. The cysteine cathepsins representing the major group of these enzymes are synthesized as inactive preproenzymes [23,24]. During the passage to the endoplasmic reticulum, the prepeptide is removed and the procathepsin undergoes proteolytic processing to the mature enzyme in the lysosomal compartment. Besides their physiological roles in cellular protein metabolism, some cysteine cathepsins are involved in several pathological processes, especially in the neuroinflammatory processes of neurodegenerative diseases represented by a tissue- and cell-specific expression pattern [25-29].

Cathepsin C (Cat C, or dipeptidyl peptidase I, DPPI), which belongs to cysteine cathepsins with a molecular mass of about 200 kDa, has been found constitutively expressed in a variety of tissues in mammals with the highest levels in lungs, kidneys, liver and spleen, but relatively low in the brain [30-34]. Cat C functions as a key enzyme in the activation of granule serine proteases in cytotoxic T lymphocytes, natural killer cells (granzymes A and B), mast cells (chymase and tryptase) and neutrophils (cathepsin G, proteinase 3, and elastase) [35-37]. The roles of Cat C and the activated serine proteases in modulating the inflammatory responses have been assessed in a number of inflammation models. For instance, Cat C knockout mice are completely resistant to the acute arthritis induced by anti-type II collagen antibodies and showed a high degree of protection in a collagen-induced rheumatoid arthritis model [38,39]. In addition, the inflammatory cells infiltration and inflammatory cytokines production have been shown to be decreased in Cat C knockout mouse models of asthma, chronic obstructive pulmonary disease (COPD), sepsis and abdominal aortic aneurysms [40-43]. Based on these studies, we hypothesized that Cat C in the central nervous system (CNS) might play a similar role in neuroinflammation. In this regard, we have found that Cat C promoted the brain demyelination in cuprizone-treated mice in our preliminary study (unpublished data). Until now, the Cat C expression has been studied only on the mRNA level in the homogenized brain tissue [32-34], the expression pattern and cellular localization of Cat C in the brain, as well as the functional role of Cat C in neuroinflammation still remain unclear.

In this study, we examined the expression pattern and cellular localization of Cat C in the brain in lipopolysaccharide (LPS)-induced neuroinflammation. We further investigated the expression, release and enzymatic activity of microglial Cat C in response to LPS and proinflammatory cytokines IL-1β, IL-6 stimulations. We found the brain Cat C expression was markedly enhanced in LPS-induced neuroinflammation, and LPS and proinflammatory cytokines could induce the expression, release and upregulate the enzymatic activity of Cat C in microglial cells.

## Materials and methods

### Animals

Eight-week-old C57BL/6 J mice weighing 25 to 30 g were used in the experiments. Animals were housed in groups of five per cage in a 22 ± 2°C and 45 ~ 65% relative humidity environment under a normal light cycle room (12-h light/12-h dark; 8:00 a.m. light on ~ 8:00 p.m. light off). All animals had free access to food and water. All procedures were in accordance with the Dalian Medical University guidelines for the proper care and use of laboratory animals and were approved by the Laboratory Animal Care and Use Committee of Dalian Medical University.

### Lipopolysaccharide-induced treatment

LPS (*Escherichia coli*, serotype 055:B5, Sigma-Aldrich Chemical Corp., St. Louis, MO. USA) was used to induce an inflammatory response. LPS was injected intraperitoneally at a dose of 5 mg/kg dissolved in sterile, endotoxin-free 0.9% saline vehicle. Control injections were equivolume vehicle. The dosage of LPS was based on a previous study of LPS-induced neurotoxicity [44].

### Tissue preparation

At the time points of one hour, six hours, twelve hours, twenty-four hours, forty-eight hours, seventy-two hours, seven days and ten days after LPS injection, mice were anesthetized with diethyl ether and perfused with 4% paraformaldehyde solution. The brains were removed and postfixed with the same fixative overnight. Brains were then cryoprotected overnight in phosphate buffered saline (PBS) containing 20% sucrose, embedded in optimal cutting temperature (OCT) compound (McCormick Scientific, St. Louis, MO, USA), and serial 18 μm sagittal sections were made with a cryostat (Leica CM 3050 S, Leica Microsystems AG, Wetzlar, Germany) and used for the immunohistochemical (IHC) and *in situ* hybridization (ISH) studies.

### *In situ* hybridization

*In situ* hybridization (ISH) was performed as described previously [45]. We used digoxigenin (DIG)-labeled Cat C (NM_009982, 193-1324 bp), IL-1β (NM_008361, 423-880 bp), TNF-α (NM_013693.2, 575-1607 bp) and inducible nitrite oxide synthase (iNOS) (NM_010927.3, 1921-2827 bp) cRNA probes. After hybridization of antisense cRNA probes, samples were incubated overnight with alkaline phosphatase-conjugated anti-DIG antibody (Roche, Basel, Switzerland) at 4°C. Color development was achieved by incubation with 4-nitro blue tetrazolium chloride/5-bromo-4-chloro-3-indolyl-phosphate (NBT/BCIP) for sixteen hours at room temperature. Some sections were counterstained with Nuclear Fast Red for observation and analysis. Others were processed for ionized calcium binding adapter molecule 1 (Iba-1) immunohistochemistry (IHC) after ISH. Images were captured using the Nikon digital camera system (DS-Fi1, Nikon Corp., Tokyo, Japan) in combination with microscopy (Nikon Eclipse 80i). The number of cells expressing IL-1β, iNOS mRNAs was counted with use of imageJ 1.41 (National Institutes of Health). Three sections from each mouse (with five mice per condition) were used for analysis.

### Immunohistochemical staining

IHC was performed as described by Ma *et al.*[46]. The following antibodies were used: mouse anti-NeuN monoclonal antibody (1:1000, Chemicon, Temecula, CA, USA), rabbit anti-GFAP polyclonal antibody (1:1000, Dako, Glostrup, Denmark), rabbit anti-Iba1 polyclonal antibody (1:500, Wako, Osaka, Japan), goat anti-Cat C antibody (1:100, R&D Systems, Minneapolis, MN, USA). Secondary antibodies were labeled with Alexa Fluor 594 (Invitrogen Corp., Carlsbad, CA, USA), Alexa Fluor 488 (Invitrogen) or biotin (1:200, Vector Laboratories Inc., Burlingame, CA, USA) antibodies. After IHC reaction, images were captured using the Nikon digital camera system (DS-Fi1) in combination with microscopy (Nikon eclipse 80i).

### Cell culture

Primary microglial cells were harvested from primary mixed glial cell cultures prepared from neonatal C57BL/6 J mice pups as previously reported [44]. In brief, after the meninges were carefully removed, the neonatal brain was dissociated by pipetting. The cell suspension was plated in 10 cm culture dish at a density of one brain per two dishes in 10 mL Dulbecco’s modified eagle medium (DMEM) (Sigma) containing 10% fetal bovine serum (FBS) (ICN Biomedicals, Aurora, OH, USA). After fourteen to twenty-one days *in vitro*, mixed glial cell cultures were dissociated by trypsinization, and the cell suspension was seeded on a petri dish and incubated for thirty minutes in a CO_2_ incubator. Adherent cells were harvested as primary microglial cells. Microglial cells were reseeded in culture plates. The purity of microglial cells was approximately 99% as determined by CD11b (rat monoclonal immunoglobulin G (IgG), clone M1/70, Abcam Inc., Cambridge, MA, USA) staining.

The immortalized murine microglial cell line BV-2 was a kind gift from Dr. XF Wu (Dalian Medical University, China) and were maintained in DMEM supplemented with 10% FBS, 2 mM glutamine and 100 U/mL penicillin/streptomycin at 37°C in 5% CO_2_ in a humidified atmosphere.

### Nitrite assay

Primary microglial cells and BV-2 cells were cultured in medium without FBS for twenty-four hours at 1.0 × 10^5^ cells/24-well plate, then treated with LPS (10 ng/mL), IL-1β (0.1 ng/mL) or IL-6 (0.1 ng/mL) for six hours, respectively. After treatment, the nitrite in the culture medium was measured as an indicator of nitric oxide (NO) production. An aliquot of the culture medium was mixed with a volume of Griess reagent (Beyotime Institute of Biotechnology, Jiangsu, China), and the absorbance at the wavelength 540 nm was determined using a microplate reader (iMark, Bio-Rad Laboratories, Tokyo, Japan). Sodium nitrite at the concentrations of 0 to 100 μM was used as a standard to assess nitrite concentrations.

### Enzyme-linked immunosorbent assay (ELISA)

Primary microglial cells and BV-2 cells were cultured in the same condition as above, prior to treatment with various concentrations of LPS (10, 50, 100 ng/mL), IL-1β (0.1, 1, 10 ng/mL), IL-6 (0.1, 1, 10 ng/mL) for six hours, respectively. After treatment, mouse Cat C ELISA kit (R&D) was used for the quantitative measurement of Cat C in the cell lysates and culture medium.

### Semi-quantitative reverse transcription polymerase Chain reaction (RT-PCR) analysis

The treatment of BV-2 cells was the same as that in ELISA. After treatment, total RNA from cells was extracted using Trizol reagent (Invitrogen) according to the manufacturer’s protocol. Reverse transcription was performed using SuperScript II Reverse Transcriptase (Invitrogen). Primers sequences: Cat C: forward: 5’ CAGAGGCCACACAGCTATCA 3’, reverse: 5’ GCATGATTTGTCAGCTCGAA 3’ (891 bp); β-actin: forward: 5’ ATCATGTTTGAGACCTTCAACA 3’, reverse: 5’ CATCTCCTGCTCGAAGTCTA 3’ (318 bp). All primers were synthesized by Takara Biotechnology Co. Ltd. (Dalian, China). The PCR profile consisted of an initial melting step of one minute at 95°C, followed by thirty cycles of thirty seconds at 95°C, fifteen seconds at 56°C and forty-five seconds at 72°C, and a final elongation step of one minute at 72°C. PCR products were separated by 1.2% agarose gel eletrophoresis. Gels were stained with ethidium bromide and analyzed under ultraviolet light. The level of Cat C gene expression was calculated as the gray ratio of PCR product of Cat C over the internal standard (β-actin). The gray values of PCR product bands were obtained from a computerized image analysis using Gel-pro Analyzer version 4.5 software (Media Cybernetics, Silver Spring, MD, USA).

### Cathepsin C enzymatic activity assay

BV-2 cells were cultured in the same condition as above, and then treated with LPS (10, 50 ng/mL), IL-1β (0.1, 10 ng/mL), IL-6 (0.1, 10 ng/mL) for six hours, respectively. Cat C enzymatic activity in cell lysates and culture medium was determined by cellular Cat C assay kit (Genmed Scientifics Inc., Arlington, MA, USA). The procedures were performed according to manufacturer’s protocols. All samples (50 μg Cat C protein/well in 96-well plate) were incubated with the substrate, H-Gly-Phe-p-Nitroaniline, at 37°C for six hours. The product formed was detected by measuring the absorbance at the wavelength 405 nm with microplate reader (iMark, Bio-Rad). Data were presented as relative folds to untreated control.

### Statistical analysis

Data were expressed as mean + standard error of the mean (SEM) from three independent experiments. All statistical analyses were performed using the Statistical Package for Social Sciences (Version 11.5). The data were evaluated for statistical significance with paired *t* test. *P* < 0.05 was considered statistically significant.

## Results

### Systemic lipopolysaccharide injection induced the neuroinflammation

**Figure 1:**
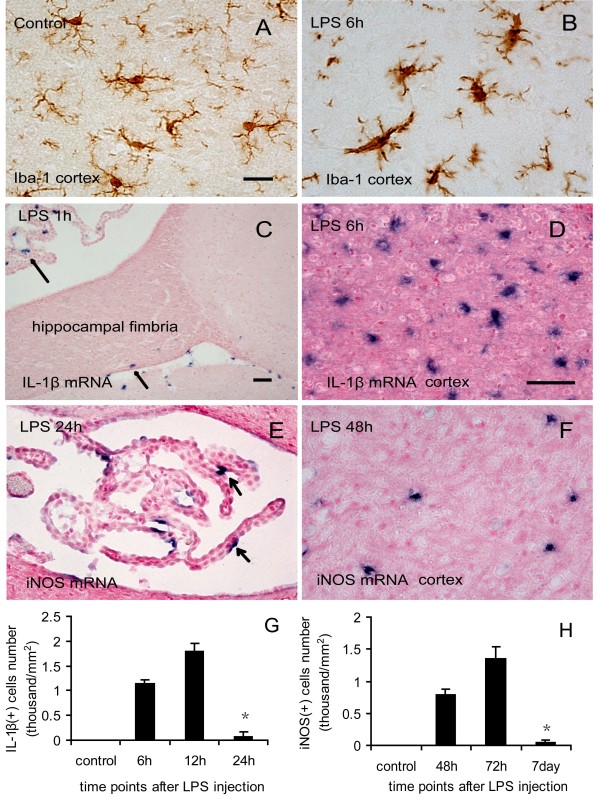
**Activation of microglial cells and the expression of proinflammatory mediators following LPS injection.** Iba-1 IHC staining showed the processes of microglial cells are ramified in control **(A)**. At six hours after LPS injection the processes of microglial cells became thicker and shorter, and cell bodies became larger **(B)**. ISH results showed IL-1β mRNA expression appeared in choroid plexus and hippocampal fimbria at one hour after LPS injection (arrows in **C**), and occurred extensively in the whole brain at six hours after LPS injection, here is an example in cerebral cortex **(D)**. iNOS mRNA expression appeared only in choroid plexus at twenty-four hours after LPS injection (arrows in **E**), and then developed in the cerebral cortex **(F)**. Quantitative analysis of the number of IL-1β mRNA- **(G)** and iNOS mRNA- **(H)** expressing cells in the cerebral cortex at different time points after LPS injection. *P < 0.05, compared with six hours, twelve hours (G); forty-eight hours, seventy-two hours (H). Scale bar = 20 μm (A, B, F); 50 μM (C); 50 μM (D, E). n = 5 per condition. Cat C, cathepsin C; Iba-1, ionized calcium binding adaptor molecule-1; IHC, immunohistochemistry; iNOS, inducible nitric oxide synthase; ISH, *in situ* hybridization; LPS, lipopolysaccharide.

In our study, a single intraperitoneal injection of LPS (5 mg/kg) did not result in death, but caused apparent systemic inflammation reflected by a fever and hepatosplenomegaly after LPS injection. To investigate whether LPS-evoked systemic inflammation affected the brain, we first examined the behavior of microglial cells by detection of morphology after systemic administration of LPS. Iba-1 (ionized calcium binding adaptor molecule-1) was used as a specific marker for microglial cells and monocytes. The IHC staining (Figure 1A and B) showed that compared to control mice (Figure 1A), Iba-1 positive cells displayed thicker and shorter processes, markedly enlarged cell bodies at six hours after LPS injection (Figure 1B). The apparent alterations of Iba-1 positive cells indicate the sufficient activation of microglial cells in the brain by systemic LPS injection, which was present for seven days after LPS injection.

To investigate whether LPS systemic injection causes inflammatory mediators release in the brain, we next examined the expressions of proinflammatory cytokines IL-1β and TNF-α mRNAs in the brain at the different time points following LPS injection by using ISH. At the same time, we also examined the level of inducible nitric oxide synthase (iNOS) mRNA expression, since nitric oxide (NO) production has been widely regarded as representative of inflammatory activation [47,48], and iNOS is responsible for generating high levels of NO in activated macrophages/monocytes. We found LPS-induced expressions of IL-1β, TNF-α mRNAs, as well as iNOS mRNA were different in temporal and spatial patterns (Figure 1C to H). At one hour after LPS injection, the expressions of IL-1β (Figure 1C) and TNF-α mRNAs (data not shown) appeared in the choroid plexus, the circumventricular organs, and the leptomeninges. At six hours, the expressions of these cytokines increased dramatically (Figure 1D and G). Peak cytokines were captured at twelve hours after LPS injection and distributed throughout the entire brain (Figure 1G). At twenty-four hours, the expression of L-1β mRNA (Figure 1G) or TNF-α mRNA (data not shown) decreased significantly and became barely detectable, compared to the expression at six hours and twelve hours. While iNOS mRNA expression started to appear in choroid plexus (Figure 1E) at twenty-four hours after LPS injection. The iNOS mRNA expression was detected in the brain parenchyma (Figure 1F) and throughout the brain at forty-eight hours and maximized at seventy-two hours. On the seventh day after LPS injection, iNOS mRNA expression decreased significantly compared to the expression at forty-eight hours and seventy-two hours (Figure 1H). Our data indicate that induced IL-1β and TNF-α mRNAs were expressed earlier than iNOS mRNA, however, the iNOS mRNA expression lasted longer than IL-1β and TNF-α mRNAs after LPS injection. The presence of highly activated microglial cells and the synthesis of proinflammatory mediators in the brain demonstrated that systemic LPS injection indeed induced neuroinflammation.

### Neuroinflammation-induced cathepsin C expression in the brain

**Figure 2:**
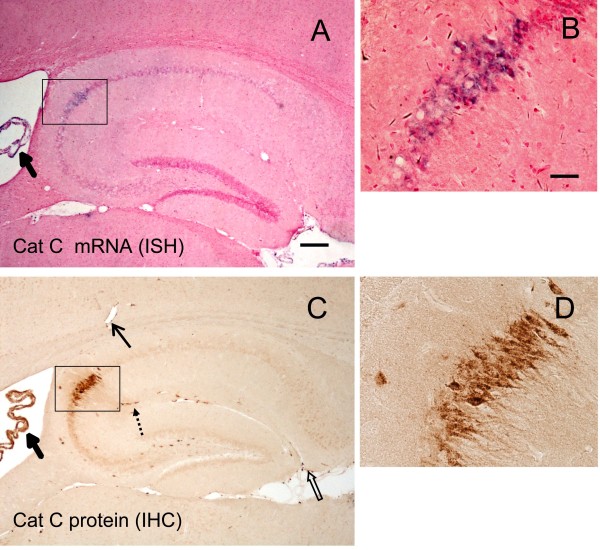
**The expression pattern of Cat C mRNA and protein in the control brain.** ISH (**A** and **B**) and IHC (**C** and **D**) staining were performed in control mice. The rectangles in A and C which included the CA2 area were enlarged into B and D, respectively. Cat C gene was predominantly expressed in the CA2 neurons of the hippocampus (B and D), interneurons (dotted arrow in C), choroid plexus (thick solid arrows in A and C), leptomeninges (open arrow in C) and vascular cells (thin solid arrow in C). Scale bar = 100 μm (A, C); 20 μM (B, D). n = 5 per condition. Cat C, cathepsin C; IHC, immunohistochemistry; ISH, *in situ* hybridization.

Previous studies have shown that Cat C plays crucial roles in the processes of peripheral inflammation, but the expression pattern of Cat C in the CNS has not been reported. Thus, first, we examined the expression pattern of Cat C in control mice by using IHC staining and ISH staining. We found that Cat C IHC staining was predominantly expressed in the pyramidal neurons of the hippocampal CA2 region (Figure 2C to D), the choroid plexus in ventricles (Figure 2A and C), the leptomeninges (Figure 2C) and the vascular cells (Figure 2C). A few Cat C-expressing neurons in the vicinity of the pyramidal neuron layer in the CA2 region appeared to be interneurons (Figure 2C). Dense Cat C-labeling granules scattered in the cytoplasm and processes, sparing the nucleus (Figure 2D). No obvious Cat C labeling was found in other brain regions. The ISH results showed that the expression pattern of Cat C mRNA was consistent with that of Cat C protein (Figure 2A to B). These findings indicate that the Cat C gene is expressed at low level in the brain under physiological conditions.

**Figure 3:**
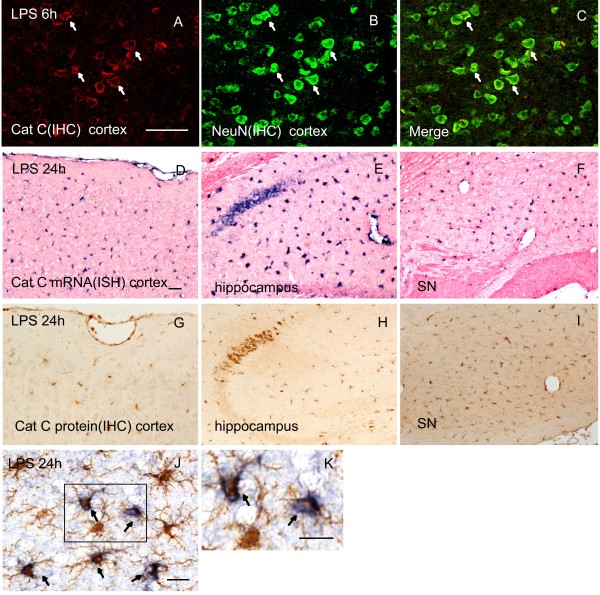
**The expression of Cat C was induced in the brain at six hours and twenty-four hours following LPS injection.** IHC double staining showed that the Cat C expression was induced in cortical neurons at six hours after LPS injection (**A** to **C**). After LPS injection for twenty-four hours, the expression of Cat C mRNA and protein was induced further and distributed throughout the entire brain, such as the cortex (**D** and **G**), hippocampus (**E** and **H**) and SN (**F** and **I**). Double staining of Iba-1 (IHC) and Cat C (ISH) showed that Cat C expression was induced in microglial cells at twenty-four hours after LPS injection (**J** and **K**). Scale bar = 20 μm (A to C, J and K); 50 μm (D to I). n = 5 per condition. Cat C, cathepsin C; Iba-1, ionized calcium binding adaptor molecule-1; IHC, immunohistochemistry; ISH, *in situ* hybridization; LPS, lipopolysaccharide. SN, substantia nigra.

Next, we tested Cat C expression in the brain by examining regional distribution and cellular localization of Cat C at different time points following LPS injection. At six hours after LPS injection, in addition to the previously reported Cat C positive cells in the CA2 region of the hippocampus under physiological conditions, a large number of Cat C immunopositive cells with neuronal morphology were found to distribute evenly in the cerebral cortex (Figure 3A). At twenty-four hours after LPS injection, we detected Cat C immunopositive signals in the cells with glial morphology in the hippocampus, cortex, substantia nigra (SN), striatum, cerebellum, brain stem and other brain regions (Figure 3G to I). Thus, we concluded that LPS injection for twenty-four hours could induce a global expression of the Cat C immunopositive signals, presumably in both neuronal and glial cells.

To confirm the phenotype in Cat C immunopositive cells, we performed double stainings in the following combination: Cat C (IHC) and NeuN (neuronal marker, IHC); Iba-1 (microglial cell marker, IHC) following Cat C (ISH); GFAP (astrocyte marker, IHC) following Cat C (ISH). At six hours after LPS injection, almost all the Cat C positive cells in the cortex expressed NeuN (Figure 3A to C), while at twenty-four hours, the majority of Cat C mRNA positive cells were colabeled with Iba-1 (Figure 3J and K), but we did not find colocalization of Cat C mRNA and GFAP (data not shown). These data indicate that six hours after LPS injection Cat C expressed in cortical neurons; twenty-four hours after, microglial cells initiated to express Cat C. We found neuronal Cat C expression became faint on the seventh day, undetectable on the tenth day after LPS injection (data not shown). Microglial Cat C expression decreased significantly and became almost undetectable at seventy-two hours after LPS injection (data not shown). These observations suggest that the time course of Cat C expression in neurons and microglial cells is different.

To determine whether the transcriptional level of the Cat C gene corresponds to the reported expression of Cat C protein in the brain, we used ISH to examine the levels of Cat C mRNA expression at same time points following LPS injection. ISH results showed alterations in the expression pattern of Cat C mRNA were similar to those of the Cat C protein (Figure 3D to F).

Taken together, the data indicate that LPS-induced neuroinflammation triggered a transient induction of Cat C expression in neurons and microglial cells with differential temporal and spatial patterns.

### Lipopolysaccharide and proinflammatory cytokines induced the expression of cathepsin C gene *in vitro*

In our present study, we systemically administrated a high-dose LPS (5 mg/kg) to mimic deleterious systemic infections. LPS administration activated microglial cells, induced proinflammatory mediators, Cat C mRNA and protein expressed in the brain. Apart from that, we also noticed that peak of IL-1β, IL-6 and TNF-α mRNAs expressions appeared at twelve hours after LPS injection, while significantly higher Cat C expression in microglial cells was detected at twenty-four hours after LPS injection. This time difference in expression suggests that proinflammatory cytokines might induce Cat C expression in microglial cells. Furthermore, Rao *et al.*[32] previously reported that the treatment of lymphocytes with interleukin-2 (IL-2) resulted in a significant increase in Cat C mRNA levels by Northern analyses. Therefore, we hypothesized an inducible effect of proinflammatory cytokines on Cat C expression in the microglial cells.

To test our hypothesis, we examined the NO concentration in the culture medium and Cat C protein and mRNA expression in cell lysates and culture medium of the BV-2 cells or primary microglial cells treated with various concentrations of LPS (10, 50, 100 ng/mL), IL-1β (0.1, 1, 10 ng/mL) or IL-6 (0.1, 1, 10 ng/mL). We first determined the NO level in the culture medium following treatment with LPS (10 ng/mL), IL-1β (0.1 ng/mL) or IL-6 (0.1 ng/mL). There was a significantly higher level of NO in the culture medium of both BV-2 cells and primary microglial cells after treatments, compared to untreated cells (*P* < 0.01) (data not shown). This indicates that these inflammatory stimulators successfully activated microglial cells, even at the lowest concentrations.

**Figure 4:**
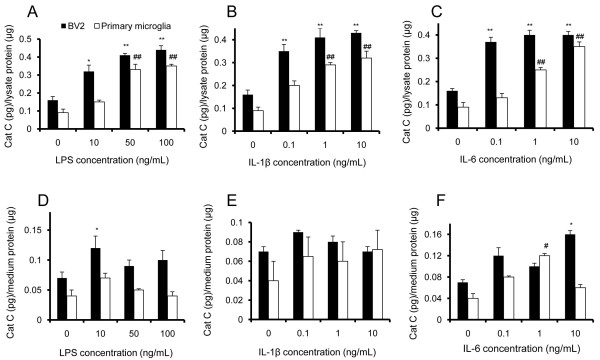
**The expression and release of Cat C were induced following LPS, IL-1β and IL-6 treatments in BV-2 cells and primary microglial cells.** The levels of Cat C in cell lysates and culture medium were measured by ELISA. The expression levels of Cat C in lysates of BV-2 cells and primary microglial cells were showed at six hours after treatment with LPS (**A**, 0 to 100 ng/mL), IL-1β (**B**, 0 to 10 ng/mL) and IL-6 (**C**, 0 to 10 ng/mL), respectively. The levels of Cat C in the medium of BV-2 cells and primary microglial cells after the same treatments were showed in **D**, **E** and **F**, respectively. * or #: *P* < 0.05, ** or ##: *P* < 0.01, compared with untreated control. Data were expressed as mean + SEM from three independent experiments. Cat C, cathepsin C; ELISA, enzyme-linked immunosorbent assay; LPS, lipopolysaccharide.

Then we examined Cat C protein levels in cell lysates and culture medium by ELISA. Compared to untreated cells cultured, Cat C protein expression was increased in a dose-dependent manner, in either BV-2 cell lysates or primary microglial cell lysates, following six-hour LPS treatment (Figure 4A). Similar results were seen in cell lysates with IL-1β or IL-6 treatment (Figure 4B and C). In contrast, the secretion of Cat C in the medium was affected to various extents (Figure 4D to F).

**Figure 5:**
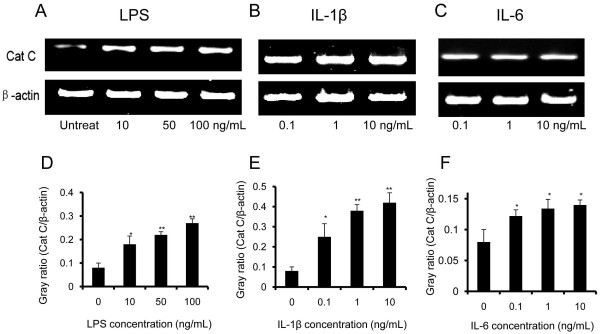
**The expression of Cat C mRNA was induced following LPS, IL-1β and IL-6 treatments in BV-2 cells.** The levels of Cat C mRNA were measured by RT-PCR after treatments of LPS (0 to 100 ng/mL), IL-1β (0 to 10 ng/mL) and IL-6 (0 to 10 ng/mL). The example of PCR product band and averaged gray ratio at the each of treatments were shown in **(A)** and (**D)**, **(B)** and **(E)**, **(C)** and **(F)**, respectively. Note a dose-dependent expression of Cat C mRNA was found in three groups of treatments. *: *P* < 0.05, **: *P* < 0.01, compared with untreated control. Data were expressed as mean + SEM from three independent experiments. Cat C, cathepsin C; LPS, lipopolysaccharide; RT-PCR, reverse transcription polymerase chain reaction.

Next, Cat C mRNA expression in stimulated BV-2 cells was measured by semi-quantitative RT-PCR, which corresponds with Cat C protein increases in microglial cells. We saw lower Cat C mRNA expression in untreated cells (Figure 5A), while a significantly higher and dose-dependent expression was seen in LPS (Figure 5A and D), IL-1β (Figure 5B and E) or IL-6 (Figure 5C and F) treated cells, suggesting that the expression of Cat C gene was subjected to transcriptional regulation.

Taken together, the expression and release of Cat C in response to LPS, IL-1β and IL-6 stimulation *in vitro* support the notion that in the brain, Cat C expression could also be induced by LPS and proinflammatory cytokines*.*

### Lipopolysaccharide and proinflammatory cytokines upregulated cathepsin C enzymatic activity *in vitro*

**Figure 6:**
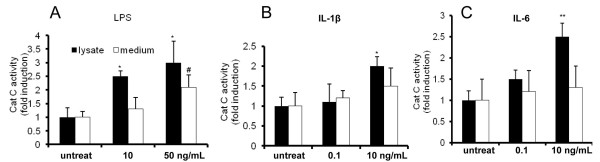
**Effects of LPS, IL-1β and IL-6 treatments on the Cat C activity in BV-2 cells.** The intracellular and extracellular activity of Cat C in BV-2 cells after six-hour treatment of LPS (10, 50 ng/mL), IL-1β (0.1, 10 ng/mL) and IL-6 (0.1, 10 ng/mL) were measured and showed in **(A)**, **(B)** and **(C)**, respectively. * or #: *P* < 0.05, **: *P* < 0.01, compared with untreated control. Data were expressed as mean + SEM from three independent experiments. Cat C, cathepsin C; LPS, lipopolysaccharide.

Cat C functions as a key enzyme in the activation of granule serine proteases in inflammatory cells. Once activated, serine proteases will trigger a series of reactions leading to tissue damage and chronic inflammation. Therefore, it is essential to investigate the alterations of Cat C enzymatic activity in neuroinflammation. In order to know whether the Cat C induced by LPS and proinflammatory cytokines has enzymatic activity, we tested Cat C activity in BV-2 cell lysates and culture medium following six-hour treatment with LPS (10, 50 ng/mL), IL-1β (0.1, 10 ng/mL), or IL-6 (0.1, 10 ng/mL). The results showed that LPS (Figure 6A), IL-1β (Figure 6B) or IL-6 (Figure 6C) significantly upregulated Cat C enzymatic activity in cell lysates, whereas the increase of Cat C enzymatic activity in the medium was only detected with LPS treatment (Figure 6A), no significant increase of Cat C enzymatic activity was found with IL-1β or IL-6 treatment (Figure 6B, C).

## Discussion

The systemic inflammation may induce neuroinflammation by producing circulating cytokines, which in return will have an important impact on the CNS [21,49]. Alterations in brain metabolism and function were observed in almost 70% of septic patients or in animals subjected to systemic inflammation or septic shock [14,50,51]. LPS, a component of the cell wall of gram-negative bacteria, could stimulate immune cells to release an array of proinflammatory mediators resulting in a septic inflammatory response [52-57]. Most effects by peripherally administered LPS are likely mediated through LPS receptors outside the BBB. LPS is a macromolecule with a molecular weight of approximately 50 to 100 kDa [58], only a minimal amount of LPS is capable to cross the BBB [59]. The mechanisms by which LPS is transduced into the CNS and causes neuroinflammation are not fully understood. Based on our observation and other studies, we considered the following possibilities: (1) Circulating LPS might enter the brain parenchyma through circumventricular organs, choroid plexus and leptomeninges. These structures which are enriched with the LPS receptor, CD14 and toll-like receptor 4 (TLR4) [60], will allow a rapid transcription of proinflammatory cytokines across the brain parenchyma. We have consistently observed the expressions of IL-1β, TNF-α in these structures at one hour after systematic LPS injection, supporting this possibility. (2) Circulating LPS and proinflammatory cytokines together act on the brain microvasculature leading to the transcriptional activation of a wide range of proinflammatory cytokines and chemokines [61], which may cross the BBB to mediate central effects. (3) Circulating LPS may enter the brain directly through opening of the BBB, but only in cases of brain infections or deleterious systemic infections associated with the opening of the BBB. In our present study, we administrated LPS at a dose of 5 mg/kg, which was reported to induce serious systemic infection [44]. It has been reported that astrocytes were severely damaged by LPS in the SN [62]. Since astrocytes are a structural component of the BBB, the permeability of the BBB could be increased due to the damaged astrocytes. (4) The inflammatory cells of the bloodstream activated by systemically administrated LPS may enter the brain and cause neuroinflammation.

Much evidence suggests that LPS-induced systemic inflammation can activate microglial cells (resident innate immune cells) and neurons in the brain [34,63,64]. And the activated microglial cells and neurons could release cathepsins from their lysosomes, resulting in the neuronal death and neurodegeneration [15-21]. Cat C is the physiological activator of serine proteases from immune and inflammatory cells vital for defense of an organism. Dominant-negative mutations of human Cat C gene are linked to the Papillon-Lefèvre syndrome [65], a disease characterized by early periodontitis, palmoplantar hyperkeratosis, and a predisposition to bacterial infections. This indicates that Cat C plays an important role in the immune system. Cat C is differentially expressed in human tissues. Northern blot analysis showed transcripts of Cat C were barely detectable in the brain [32-34]. In our study, ISH and IHC staining revealed the expression of Cat C mRNA and protein was restricted to the hippocampal CA2 region, choroid plexus, leptomeninges and vascular cells in control mice. To the best of our knowledge, it is the first report of expression pattern of Cat C in the CNS under physiological conditions. However, the functional significance of specific Cat C expression pattern in the pyramidal neurons of hippocampus still remains unclear.

In our present study, at six hours after LPS injection, a substantial Cat C expression was found in cortical neurons, the expression was present for ten days, but these neurons did not change the least bit in morphology. These results indicate that a single LPS injection is not sufficient to cause obvious damage to neurons. Furthermore, the Cat C mRNA and protein expressions were detected in microglial cells throughout the entire brain at twenty-four hours after LPS injection and were present for up to three days, implying a differential time-related regulation of Cat C expression in neurons and microglial cells in the brain in response to systemic immune challenges. Previous studies reported that several serine proteases expressed in neurons and glial cells, such as thrombin, trypsin-like serine proteases, are involved in modulating neuroinflammation and neuroprotection events in the CNS [66-68]. However, the precise mechanisms for activating these enzymes in brain tissue still remain obscure. Since Cat C is capable of activating many serine proteases in peripheral tissues, Cat C is likely to be a good candidate for activating brain serine proteases. In our present study, we cannot define whether the functional significance of an increased Cat C expression in a neuron is a transiently protective response or a detrimental response to acute inflammation.

In this study, using cell lines and primary cells, we further determined inflammatory factors involved in induction of microglial Cat C expression and enzymatic activity. We found the Cat C mRNA and protein expressions in microglial cells were increased dose-dependently upon various inflammatory stimulations (LPS, IL-1β and IL-6)*.* Also, these inflammatory mediators increased the enzymatic activity of Cat C in microglial cells, suggesting increased Cat C activity could be due to increased mRNA and protein levels. On the other hand, LPS caused Cat C activity to increase in the culture medium, which could be attributed to increased secretion of active Cat C from microglial cells. Our study suggests regulation of expression, secretion and activity of Cat C in microglial cells by various inflammatory stimulations that may be present *in vivo* under various circumstances, for example, CNS injury and nerve degeneration [69-71].

Cat C is synthesized as inactive preprocathepsin C *in vivo* and is processed into its active mature form by a series of proteolytic cleavages. Some endopeptidases, presumably cathepsin L or S, are responsible for the activation of Cat C [72]. Once activated, Cat C mediates the conversion of granule serine proteases from their inactive (zymogen) into the enzymatically active protease by removing an N-terminal propeptide [38]. Cat C plays an immunoregulatory role through activating serine proteases during inflammation. The findings in Cat C knockout mice supported the role of Cat C in modulating inflammatory processes *in vivo.* Neutrophils of Cat C knockout mice showed very little enzymatic activity of serine proteases and demonstrated a strongly decreased infiltration to the sites of inflammation in either an experimental arthritis model or an asthma model [38,43]. Importantly, this was accompanied by decreased local production of inflammatory chemokines (such as CXCL2) and cytokines (such as TNF-α, IL-1β, IL-6). Based on these studies of Cat C mediating peripheral inflammation and our present findings in CNS, we hypothesize that the functional role of Cat C in LPS-induced neuroinflammation is as follows: the activated microglial cells produce proinflammatory cytokines which may induce Cat C expression and enzymatic activity; Cat C further modulates cellular events through activating serine proteases, leading to cytokines amplification. Hence, the interaction between inflammatory cytokines and Cat C in the brain may be responsible for the continuous inflammatory cycle in neurodegenerative diseases as well as other neuroinflammation-involved neurological disorders.

## Conclusions

In our current study, we established an animal model of neuroinflammation by LPS systemic administration. We presented data showing the regional distribution and cellular localization of Cat C, a lysosomal cysteine protease, in the brain. Our study demonstrated the alterations of Cat C in neuroinflammation. We found Cat C was expressed in the brain at low level under physiological conditions. After LPS administration, Cat C expression was induced in neurons and microglial cells at different time points. *In vitro* preparations*,* stimulations of LPS and proinflammatory factors IL-1β, IL-6 induced intracellular expression and extracellular release of Cat C as well as upregulation of enzymatic activity. On the basis of these results and previous studies showing Cat C mediated peripheral inflammation, we provided cellular evidences to support the notion that Cat C could participate in the development of neuroinflammation in the CNS. Therefore, further studies of the functional role of Cat C in the mechanism of neuroinflammation in the CNS may provide an insight for the therapeutic prevention and/or treatment of neuroinflammation-involved neurological disorders in the future.

## Abbreviations

BBB, Blood–brain barrier; BCIP, 5-bromo-4-chloro-3-indolyl-phosphate; Cat C, Cathepsin C; CNS, Central nervous system; DIG, Digoxigenin; DPPI, Dipeptidyl peptidase I; DMEM, Dulbecco’s modified eagle medium; ELISA, Enzyme-linked immunosorbent assay; FBS, Fetal bovine serum; IHC, Immunohistochemistry; IL, Interleukin; iNOS, Inducible nitrite oxide synthase; ISH, In situ hybridization; kDa, KiloDaltons; LPS, Lipopolysaccharide; mAb, Monoclonal antibody; NBT, 4-nitro blue tetrazolium chloride; NO, Nitrite oxide; PBS, Phosphate buffered saline; RT-PCR, Reverse transcription polymerase chain reaction; SEM, Standard error of the mean; SN, Substantia nigra; TNF-α, Tumor necrosis factor-α.

## Competing interests

The authors declare that they have no competing interests.

## Authors’ contributions

JM conceived of the study, participated in its design and coordination and helped to draft the manuscript. FK participated in the design of the study, carried out the immunoassays, RT-PCR *in vitro* and drafted the manuscript. XF carried out enzymatic activity determination *in vitro.* FB and LN carried out the experiments *in vivo.* YZ carried out cell culture. YW performed the statistical analysis. All authors read and approved the final manuscript.

## Authors’ information

Jianmei Ma, PhD and MD, professor, Department of Anatomy, Dalian Medical University, major in neuroscience, especially in molecular mechanism of demyelinating disease in CNS.

Kai Fan, PhD and MD, associate professor, Department of Anatomy, Dalian Medical University, major in neuroinflammation-involved neurological disorders.
